# Right femoral pathological fracture caused by primary bone epithelioid angiosarcoma: Case report

**DOI:** 10.1097/MD.0000000000006951

**Published:** 2017-07-07

**Authors:** Yatong Li, Xiongfei Zou, Xiaoyan Chang, Xiao Chang, Shengfang Sun, Baozhong Zhang

**Affiliations:** a Department of Orthopedics; b Department of Pathology, Peking Union Medical College Hospital, Beijing; c Department of Emergency Surgery, Affiliated Hospital of Binzhou Medical College, Shandong, China.

**Keywords:** epithelioid angiosarcoma, imaging examination, multiple bone destruction, pathological fracture

## Abstract

**Rationale:**

Epithelioid angiosarcoma (EAS) is an extremely rare malignant disease, which accounts no more than 1% of all soft tissue sarcomas. In this article, we would report a new case of EAS with multiple bone destruction and right femoral pathological fracture, which was an even rarer manifestation of EAS.

**Patient concerns:**

In this case, a 64-year-old man with right femoral fracture was reported. He had suffered from a progressive low back pain for about 8 months, and the imaging examinations prompted a multiple bone destruction in his vertebra and lower limbs. He then got a right femoral fracture without any obvious traumatic injury, and came to our hospital.

**Interventions:**

He underwent an operation of radical resection, bone cement filling and dynamic condylar screw internal fixation. During the operation, we found that the soft tissue around the fracture had a rotten fish change, which suggested a malignant disease.

**Diagnoses:**

The postoperative pathological diagnosis reported an EAS, which is extremely rare and highly malignant.

**Outcomes:**

The patient died in 83 days after the surgery, and the survival time from the symptoms started to the end was only 11 months, which showed a rapid progress and poor prognosis of EAS.

**Lessons:**

EAS is very hard to be diagnosed by clinical manifestation or radiological examinations. As in our case, pathological analysis is the final diagnosis. The images of the patient may offer some tips for the skeletal presentation of EAS, and do more help in future study of this disease.

## 1 Introduction

Epithelioid angiosarcoma (EAS) is an extremely rare malignant tumor.^[1–3]^ It is a rare sub-type of angiosarcoma, which accounts only 2% of all soft tissue sarcomas.^[4]^ EAS could lead to many different lesions, such as thyroid gland, skin, ureter, adrenal gland, kidney, small bowel, and so on. However, there were only a few literature about its skeletal involvement, especially as the primary lesion. Thus, we would report a new case about multiple bone destruction and pathological fracture caused by EAS, to discuss the skeletal manifestation of this disease.

## 2 Case presentation

In September 2016, a 64-year-old man presented to us with a chief complaint of right thigh pain, swelling, and activity limitations for 1 week. Actually, he had suffered from a continuous progressive low back pain for about 8 months, and the pain impeded his daily life seriously, with the visual analogue scoring system (VAS) score evolving from 3 to 7. One week ago, he felt a right thigh sharp pain suddenly without any inducement or traumatic injury, with a VAS score 9. The swelling emerged immediately and all the activities of his right leg were restricted completely. The patient denied any thoracic or abdominal discomfort since onset, and there was no obvious body weight change. The past history, personal history, and family history were nothing special.

**Figure 1:**
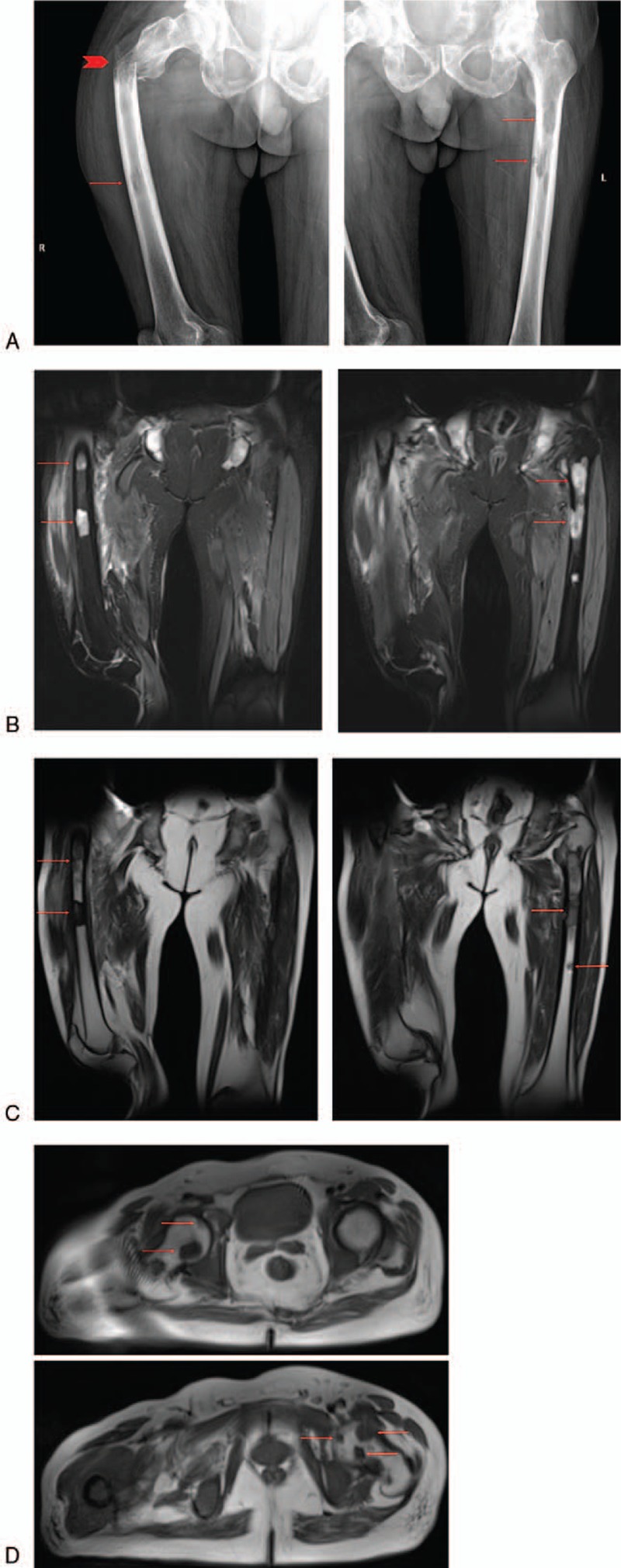
The x-ray (A) and MRI examination (B–D) of the patient's femurs, showed a pathological fracture (the arrowhead) on the right femur with multiple low-density lesions of both femurs, which were bone destruction (the arrows). MRI = magnetic resonance imaging.

**Figure 2:**
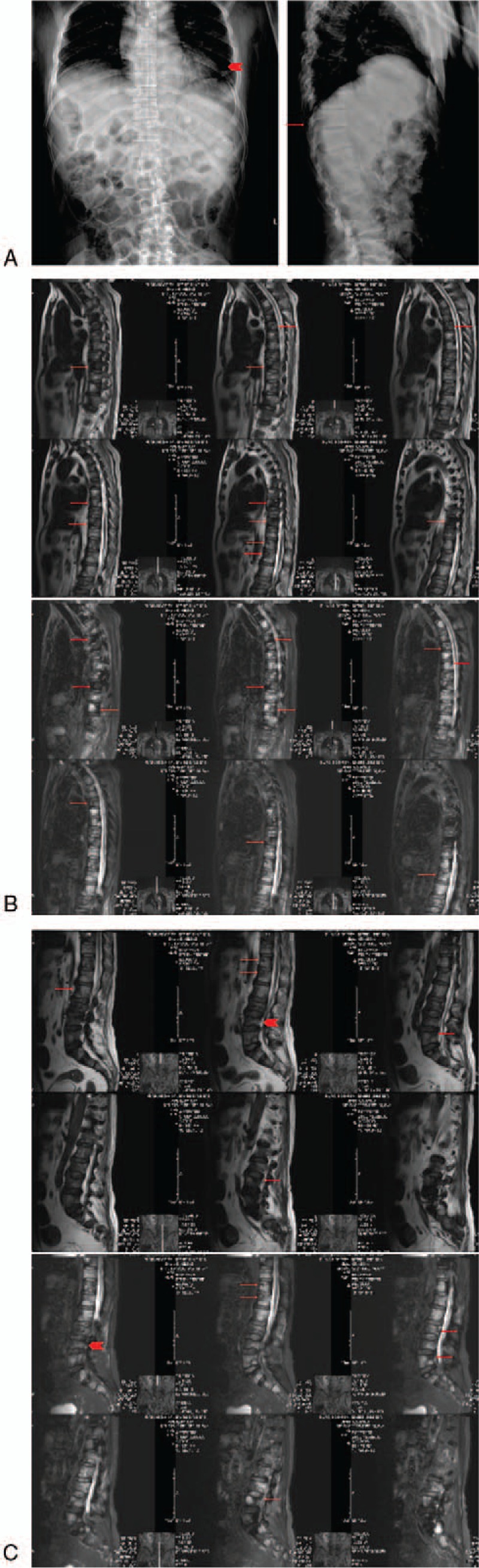
The imaging examination of the patient's ribs (A), thoracic vertebra (B), and lumbar vertebra (C) showed a multiple pathological fractures (the arrowheads) and bone destruction (the arrows).

**Figure 3:**
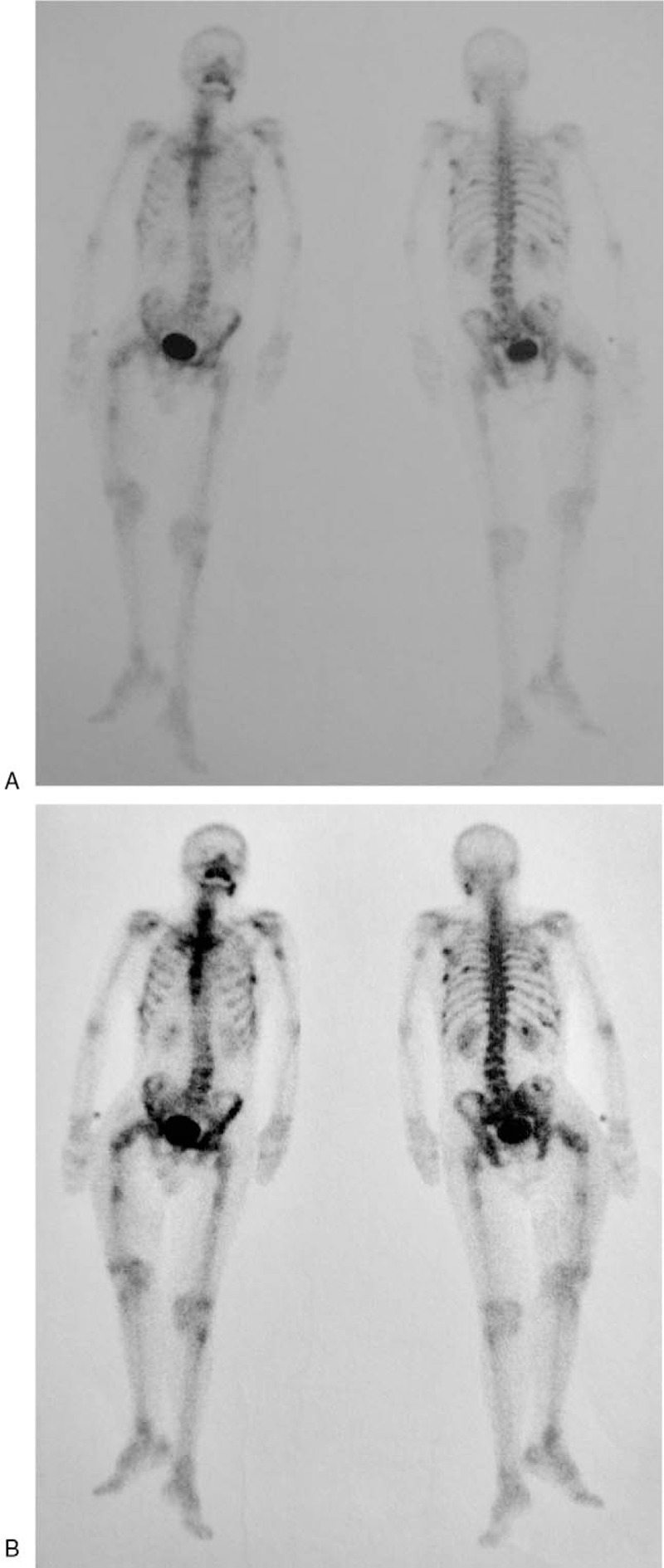
The bone scanning examination of the patient before (A) and after (B) the tracer injection, showed a multiple bone destructions all over the body.

The x-ray examination showed a right proximal femoral fracture, with multiple low-density areas in bilateral femoral bone marrow cavity (Fig. 1A). The magnetic resonance image (MRI) examination reported the same results (Fig. 1B–D). Multiple abnormal segmental signals in bone marrow strongly suggested malignant changes, or metastasis. The similar bone destruction and multiple pathological fractures could also be seen in the ribs, thoracic vertebrae, and lumbar vertebrae (Fig. 2). The bone scanning examination and positron emission tomography-computerized tomography (PET-CT) showed multiple bone destruction all over the body (Fig. 3), as well as limited atelectasis and hepatic cyst.

Multiple myeloma was first considered, while bone metastasis of small cell lung cancer was also very possible, since the patient's neuron specific enolase and pro-gastrin-releasing peptide levels were higher than normal (17.6 and 79.4 pg/mL, respectively). However, the bone marrow needle biopsy result did not support the diagnosis of multiple myeloma. The proportion of neutrophilic segmented granulocyte was higher, up to 40.0%, while that of erythrocytic series was lower. The ratio of lymphocytes and monocytes was within the normal range, and the shapes of all kinds of blood cells were normal. At mean time, the thoracic computerized tomography and PET-CT did not report any pulmonary or mediastinal occupying lesions, which made the diagnosis of small cell lung cancer uncertain. Thus, the primary cause of the pathological fracture became confusing and interesting.

**Figure 4:**
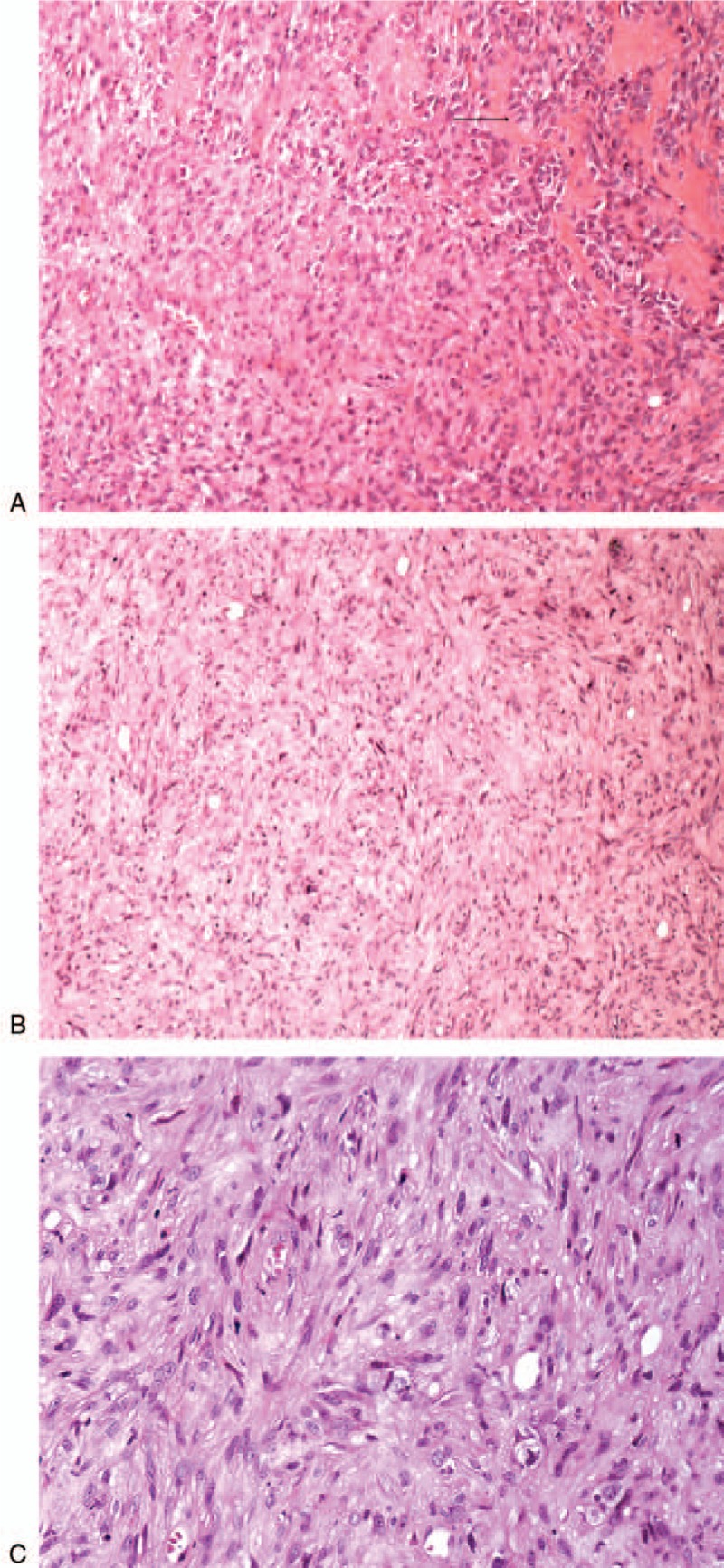
The pathological diagnosis of the gelatinous pus-like tissues of the right femoral fracture: EAS. (A) Hematoxylin-eosin staining of the mixture of tumor cells (the arrow) and fractured broken bone, 150×; (B) Hematoxylin-eosin staining of tumor cells, 60×; (C) Hematoxylin-eosin staining of those irregular tumor cells, 200×. EAS = epithelioid angiosarcoma.

The patient underwent an operation of radical resection, bone cement filling, and dynamic condylar screw internal fixation on his right femur. During the procedure, we found that the soft tissue around the fracture had a rotten fish change, while the bone marrow cavity was filled with some gelatinous pus-like tissues and blood, which certainly suggested a malignant disease and a poor prognosis. The postoperative pathological analysis diagnosed it as EAS, which is highly malignant, and extremely rare (Fig. 4).

The patient gave his informed consent for treatment and inclusion in this study, after having been provided with all the necessary information. During a 2-month follow-up, the patient's right leg had a good recovery. However, he could still feel pain in his back and left thigh, with a VAS score 6 to 7. The patient died in 83 days after his surgery before he started to accept any postoperative comprehensive treatments. The survival time from the symptoms started to the end was only 11 months, showed a rapid progress of this rare malignant disease.

## 3 Discussion

Angiosarcoma is an uncommon malignant tumor. It is derived from vascular tissues and accounts for only 2% to 4% of all soft tissue sarcomas.^[5–9]^ It could develop in any sites, including the skin, soft tissues, breast, liver, and spleen, and represent only 1% of primary malignant neoplasms in bone or marrow.^[10]^ The mechanism of angiosarcoma is still unknown, while the related risk factors include previous exposure to radiation or some special chemotherapy for lymphoma.^[11,12]^ Angiosarcoma is not easy to be diagnosed just by clinical and radiological features. Thus, postoperative pathological examination remains the gold standard.^[4,9,13]^ Comparing with the similar diseases such as hemangioma or haemangioendothelioma, which are benign or borderline malignant neoplasms, angiosarcoma is much more malignant.^[14]^ The prognosis of angiosarcoma is very poor with a 1-year survival rate less than 50%, and the elder age, larger tumor size, and retroperitoneal location are poorer prognostic factors.^[5,15–19]^

EAS is an even rarer malignant mesenchymal tumor, which belongs to angiosarcoma. In terms of pathology, EAS could coexpress both vascular and epithelial markers, which make it named and hard to be distinguished from metastatic carcinomas. The first case about EAS of bone was reported by Dr. Balicki, etc., in 1996,^[14,20]^ but there were no more than 30 cases reported all over the world until now.^[14,20–31]^ EAS of bone could involve the femur, tibia, talus, pelvis, spine, scapula, or multiple bones, but there were too few cases to do significant analysis.^[14,20–31]^

The clinical manifestations of EAS of bone were different according to the various locations of the primary lesions, while ostealgia and activity limitations were most commonly seen.^[20–31]^ However, it was hard to say which lesion was the primary one when EAS had already made multiple bone destruction at the diagnostic time just like our case. Since the extremely malignant features of EAS, there were no absolutely standard treatments. Surgical treatment for local lesion with postoperative radiotherapy and chemotherapy was suggested to be effective, but sometimes it was just palliative treatment.^[32]^

Under the microscope, EAS is described to have large, rounded malignant cells with epithelioid features, as well as plentiful amphophilic or eosinophilic cytoplasm, and round to irregular vesicular nuclei with variably accentuated nucleoli (Fig. 4A and B). EAS cells are high-grade tumor cells and appear to be arranged in sheets, nests, cords, or rudimentary vascular channels (Fig. 4C).^[32–34]^

Immunohistochemistry is very significant in diagnosing EAS. CD31 and CD34 are some usual vascular antigens and would be expressed by EAS in different degrees.^[9,33,35–37]^ CD31 is considered to be a high specific and sensitive endothelial marker, and it is reported to be positive in 90% of all types of AS. At the same time, the expression of CD34 in angiosarcoma remains controversial.^[1,9,35–41]^ In our case, CD31 was strongly positive expressed in those EAS tumor cells just like all kinds of vascular tumors, while CD34 was partially positive. As for the epithelial markers, cytokeratin (CK) could be positive at a rate of 30% to 50% in EAS cells, while epithelial membrane antigen seems to be negative all the time.^[42]^ In our case, cytokeratin-7 was positive, while cytokeratin-19, cytokeratin-20, and epithelial membrane antigen were all negative.

One important differentiated diagnosis of EAS is metastatic carcinoma. A higher proliferation index, some relatively typical morphological features, as well as the expression of both vascular and epithelial markers are very useful in distinguishing EAS from some other carcinomas, such as epithelioid malignant mesothelioma, epithelioid haemangioma, epithelioid haemangioendothelioma, and so on.^[18,33,41,42]^ In our case, the index of Ki-67 is about 5%, which showed a relatively more frequent mitoses and reflected a more malignant performance. A focal positive expression of anion exchanger 1/ anion exchanger 3 might indicate a metastatic malignancy, but the negative expression of thyroid transcription factor 1 and anaplastic lymphoma kinase—sub-protein 8 denied the possibility of lung cancer, colorectal cancer, and lymphomas. Considering the result of PET-CT, the possibility of multiple bone metastasis caused by some other malignancies was excluded.

The prognosis of EAS is very poor with a high rate of early metastasis and tumor-related death.^[5,9,17,19,43]^ An adequate surgery combined with the use of neoadjuvant and adjuvant chemoradiotherapy seems to improve the survival rate.^[9,43]^ However, in our case, the patient presented to us with a multiple bone destruction. It was very hard to treat all those lesions in his bone and marrow by surgery. Thus, the postoperative chemoradiotherapy became crucial and necessary. It was extremely rare that the primary manifestation of EAS was progressive ostealgia and multiple bone destruction, instead of a localized lesion. Therefore, an even poorer prognosis of this patient was predictable.^[16]^ Consequently, our case showed some tips for the skeletal presentation of EAS, and do more help in future study of this disease.

## Acknowledgments

We would like to express our gratitude to Dr. Kaidi Li, Dr. Zuoguan Chen, and Dr. Junce Jia, as well as the nurses, Yaping Chen, Lu Che, and Yuan Ma for their help in taking care of this patient.
